# Prehospital score for acute disease: a community-based observational study in Japan

**DOI:** 10.1186/1471-227X-7-17

**Published:** 2007-10-15

**Authors:** Yasuhiro Toyoda, Yoshio Matsuo, Hiroyuki Tanaka, Hidekazu Fujiwara, Toshio Takatorige, Hiroyasu Iso

**Affiliations:** 1 Public Health, Department of Social and Environmental Medicine, Graduate School of Medicine, Osaka University, Osaka, Japan; 2 Emergency Division, Kishiwada Tokushukai Hospital, Kishiwada, Japan; 3 Emergency Division, Kishiwada City Hospital, Kishiwada, Japan; 4 Kishiwada City Fire Department, Kishiwada, Japan

## Abstract

**Background:**

Ambulance usage in Japan has increased consistently because it is free under the national health insurance system. The introduction of refusal for ambulance transfer is being debated nationally. The purpose of the present study was to investigate the relationship between prehospital data and hospitalization outcome for acute disease patients, and to develop a simple prehospital evaluation tool using prehospital data for Japan's emergency medical service system.

**Methods:**

The subjects were 9,160 consecutive acute disease patients aged ≥ 15 years who were transferred to hospital by Kishiwada City Fire Department ambulance between July 2004 and March 2006. The relationship between prehospital data (age, systolic blood pressure, pulse rate, respiration rate, level of consciousness, SpO_2 _level and ability to walk) and outcome (hospitalization or non-hospitalization) was analyzed using logistic regression models. The prehospital score component of each item of prehospital data was determined by beta coefficients. Eligible patients were scored retrospectively and the distribution of outcome was examined. For patients transported to the two main hospitals, outcome after hospitalization was also confirmed.

**Results:**

A total of 8,330 (91%) patients were retrospectively evaluated using a prehospital score with a maximum value of 14. The percentage of patients requiring hospitalization rose from 9% with score = 0 to 100% with score = 14. With a cut-off point score ≥ 2, the sensitivity, specificity, positive predictive value and negative predictive value were 97%, 16%, 39% and 89%, respectively. Among the 6,498 patients transported to the two main hospitals, there were no deaths at scores ≤ 1 and the proportion of non-hospitalization was over 90%. The proportion of deaths increased rapidly at scores ≥ 11.

**Conclusion:**

The prehospital score could be a useful tool for deciding the refusal of ambulance transfer in Japan's emergency medical service system.

## Background

The Japanese national emergency medical service system has been established since 1963 and enables those in need of urgent medical treatment to summon an ambulance by calling the free national emergency telephone number '119'. Approximately 78% of ambulances are staffed by emergency life-saving technicians, who were introduced in 1991. Prior to that, emergency medical technicians played a major role in prehospital settings but were only allowed to perform basic life support procedures such as external chest compression and ventilation with a bag valve mask. Emergency life-saving technicians, by contrast, are permitted to perform endotracheal intubation, defibrillation, intravenous infusion of Ringer's solution, and administration of epinephrine. However, these treatments are allowed only for cardiopulmonary arrest patients. In addition, they are not permitted to carry out additional life-saving interventions and clinical tests such as needle thoracostomy, cricothyroidotomy, 12-lead electrocardiograms, blood glucose measurements and administration of drugs other than epinephrine. Furthermore, the frequency with which vital signs are taken at the scene is still low in Japan; 25% for blood pressure and 27% for SpO_2 _[1]. As a result, prehospital care in Japan is very limited compared with to in western countries [2-5].

Meanwhile, the Japanese national medical insurance system and free ambulance call-outs have resulted in a lack of concern over ambulance usage. This usage has increased consistently by over 5 million cases per year since 2004. According to national data, 51% of these were mild cases that did not require hospitalization [1]. In addition, the rapid aging of society, with more than 19% of the population aged over 65 years in 2004 [6], has promoted an increase in ambulance usage, especially for acute disease [7,8].

In this situation, refusal of ambulance transfer for mild cases is being debated nationally. The fire and disaster management agency established a 'Committee for demand of ambulance usage' in 2006. For appropriate ambulance usage for hospitalization, a simple triage tool to decide the refusal of ambulance usage would be useful.

The purpose of this study was to investigate the relationship between prehospital data and hospitalization outcome for acute disease patients aged ≥ 15 years, and to develop a simple tool for deciding the refusal of ambulance usage based primarily on vital signs.

## Methods

### Study setting

This study was conducted in Kishiwada City, Osaka Prefecture, Japan, which has a population of about 200,000 and an area of 72 km^2^. Kishiwada City Fire Department controls the emergency medical service system. A total of 17,293 patients were transferred by the Kishiwada City Fire Department ambulances between July 2004 and March 2006. The subjects in the present study were 9,160 consecutive acute disease patients aged ≥ 15 years during this period.

All ambulances in Kishiwada carry at least one emergency life-saving technician who records prehospital data such as patient age, vital signs, chief complaint and simple physical examinations, taken at the scene or in the ambulance. Since April 2004, prehospital data have been entered into the computer database by the captain of each ambulance, who has the emergency life-saving technician national license. Approximately 80% of emergency patients are transferred to the two main hospitals; Kishiwada City Hospital and Kishiwada Tokushukai Hospital. Five or more clinical physicians, four or more emergency nurses, one or more radiology technicians, one or more clinical laboratory technicians and one or more pharmacists reside in these hospitals over each 24-hour period. These hospitals are certificated as emergency hospitals by the Japan Acute Medicine Association.

### Study design and protocol

We investigated the relationship between prehospital data and hospitalization outcome for the acute disease patients transported by the Kishiwada City Fire Department ambulances, using a multivariable logistic regression model, and developed the prehospital score by the beta coefficients of significant variables.

The following prehospital data collected by emergency personnel, including emergency life-saving technicians, were extracted from the database: age, systolic blood pressure, pulse rate, consciousness level, SpO_2 _and ability to walk. These are routinely taken at the scene or in the ambulance and are relatively objective.

The outcome was reported as hospitalization or non-hospitalization. The chief of the emergency section of Kishiwada City Fire Department, a trained emergency life-saving technician, confirmed the hospitalization outcome and the final diagnosis by the attending physicians using FAX or telephone. Deaths in the emergency room were regarded as hospitalization. In addition, the outcome after hospitalization was confirmed for patients transferred to the two main hospitals (Kishiwada City Hospital and Kishiwada Tokushukai Hospital), i.e. 71% of the total number of subjects.

The continuous variables – age, systolic blood pressure, pulse rate and SpO_2 _– were converted to categorical variables for practical use. We decided the reference of each continuous variable according to normal clinical range. Systolic blood pressure levels were categorized as <80, 90–99, 100–149 (reference), 150–159, 160–169, 170–179, 180–189, 190–199, and ≥ 200 mmHg; pulse rates were categorized as <50, 50–59, 60–89 (reference), 90–99, 100–109, 110–119, and ≥ 120 beats per min; SpO_2 _was categorized into <95% (hypoxic) and ≥ 95% (normal). Patients with capillary circulation failure whose SpO_2 _levels could not be measured were also regarded as hypoxic. Ages were categorized as 15–59, 60–69, 70–79, 80–89 and ≥ 90 years; the consciousness level was categorized according to the Japan Coma Scale (JCS) as 0, I, II and III; the ability to walk was categorized as "yes" or "no". Cases in which the emergency life-saving technician advised the patient not to walk were regarded as "no". The major category of the Japan Coma Scale (JCS), a widely used criterion for evaluation of consciousness in Japan, was used in the present study. The scale is 0 = alert, I = awake without stimulation, II = awake with stimulation, III = not awake with stimulation [9]. When multiple data were available for each patient, the initial data were used for analyses.

The prehospital score was determined as a simple integer (0, 1, 2 or 3) for practical use on the basis of the beta coefficient for each independent variable. The total score was obtained from the sum of all items of prehospital data.

Eligible patients were retrospectively scored and the distributions of all scores for the hospitalized and non-hospitalized groups were examined. The proportion hospitalized was calculated and related to the total score. Moreover, for patients transported to the two main hospitals (Kishiwada City Hospital and Kishiwada Tokushukai Hospital), the proportions (1) non-hospitalized, (2) discharged after hospitalization, (3) transported to another hospital and (4) died in the emergency room or after hospitalization were related to the total score.

The sensitivity, specificity, positive predictive value (PPV) and negative predictive value (NPV) for predicting hospitalization were calculated at each score point, and the area under the receiver operating characteristic (ROC) curve was determined with 95% confidence intervals.

The statistical analysis package SPSS 12.0J for Windows (SPSS Japan inc., Tokyo, Japan) was used for data analyses. All p-values were two-tailed, and p-values < 0.05 were considered statistically significant.

### Ethical approval

Prehospital and outcome data did not include personal information. The present study was approved by the ethical committee of the Graduate School of Medicine, Osaka University, Japan.

## Results

### Multivariable logistic regression and development of prehospital score

From a total of 9,169 patients, complete data were available for 8,330 (91%), which were used for the analyses. Of these, 36% (3,002) were hospitalized. The mean age (± SD) of the hospitalization group was 70 ± 16 years while that of the non-hospitalization group was 58 ± 20 years.

Table 1 shows the beta coefficient and multivariable odds ratios for the components of prehospital score. All independent variables – age, systolic blood pressure, pulse rate, level of consciousness, SpO_2 _and ability to walk – were statistically significant in predicting hospital outcome.

**Table 1:** Multivariable odds ratios of hospitalization in relation to prehospital score

Prehospital data	No. of hospitalization/patients	beta coefficient	OR (95%CI)	Prehospital Score
Age, y				
<60	639/3,101	0	1	0
60–69	513/1,465	0.53	1.69(1.45–1.97)*	2
70–79	939/2,098	0.83	2.30(2.01–2.64)*	2
80–89	730/1,363	1.04	2.82(2.42–3.29)*	3
>=90	181/303	1.15	3.16(2.41–4.13)*	3
				
Systolic blood pressure, mmHg				
<80	239/277	0.93	2.53(1.63–3.94)*	2
80–89	96/185	0.55	1.73(1.25–2.41)*	2
90–99	158/428	-0.05	0.95(0.75–1.20)	0
100–149	1,489/4,512	0	1	0
150–159	236/767	-0.14	0.87(0.73–1.05)	0
160–169	223/659	-0.04	0.97(0.80–1.23)	0
170–179	134/479	-0.01	0.99(0.80–1.23)	0
180–189	134/397	-0.09	0.91(0.72–1.16)	0
190–199	81/215	0.14	1.15(0.84–1.56)	0
>=200	182/411	0.37	1.45(1.15–1.81)*	1
				
Pulse, per min				
<50	208/235	0.92	2.51(1.52–4.13)*	2
50–59	90/273	-0.03	0.97(0.73–1.29)	0
60–89	1,265/4,238	0	1	0
90–99	447/1,372	0.09	1.10(0.95–1.27)	0
100–109	366/954	0.23	1.26(1.07–1.49)*	1
110–119	245/541	0.52	1.67(1.37–2.05)*	2
120–129	218/437	0.63	1.88(1.50–2.35)*	2
>=130	163/280	0.71	2.02(1.53–2.67)*	2
				
Consciousness, JCS				
0	1,990/6,699	0	1	0
I	485/942	0.51	1.67(1.44–1.94)*	2
II	188/283	1.09	2.98(2.27–3.90)*	3
III	339/406	1.35	3.84(2.82–5.23)*	3
				
Saturation O_2_, %				
>=95	1,804/6,571	0	1	0
<95	1,198/1,759	0.99	2.69(2.36–3.06)*	2
				
Ability to walk				
yes	389/2,228	0	1	0
no	2,613/6,102	0.73	2.08(1.82–2.37)*	2

CI; Confidence Interval JCS; Japan coma scale OR; Odds ratio

P value for logistic regression analysis ; *P < 0.01

The prehospital score component was equated to 1, 2 or 3 when the beta coefficient was <0.5, 0.5–1.0 or ≥ 1.0, respectively. The total score calculated by adding the six component scores to give a value from 0 to 14.

### Results of retrospective scoring

**Figure 1:**
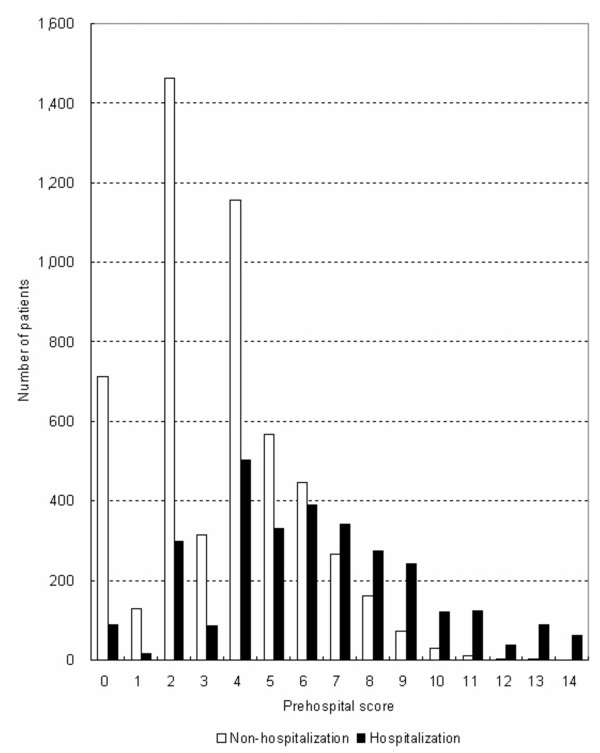
**The number of patients according to prehospital score**. The modal score of the hospitalization group was 4 while that of the non-hospitalization group was 2 (n = 8,330).

The distribution of total scores for the hospitalization and non-hospitalization groups is shown in Figure 1; the hospitalization group follows an approximately normal distribution, while the non-hospitalization group is skewed with most patients receiving a low score. The modal score of the hospitalization group was 4 while that of the non-hospitalization group was 2.

**Figure 2:**
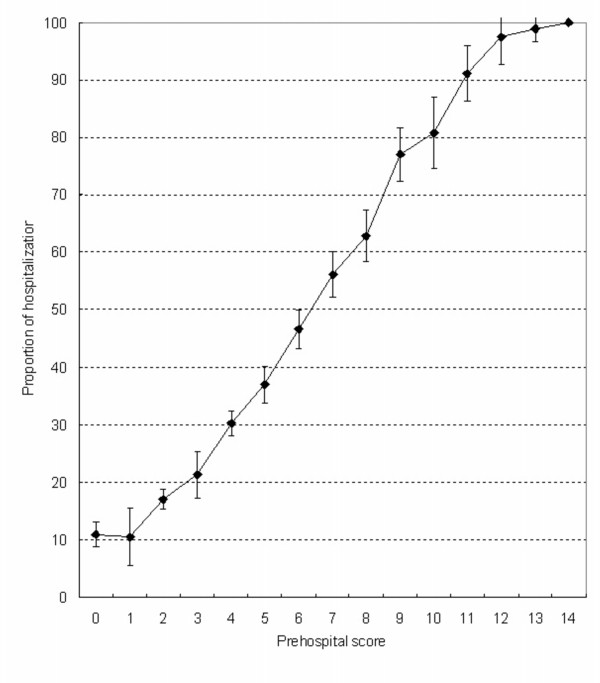
**The proportion hospitalized (with 95% confidence intervals) in relation to prehospital score**. A linear relationship was observed between the score and the proportion hospitalized (n = 8,330).

Figure 2 shows the proportion and 95% confidence interval of hospitalization in relation to total score. A linear relationship was observed between the score and the proportion hospitalized: 9% of patients with a score of 0 required hospitalization, and this increased to 100% for those with a score of 13.

Among the 102 patients requiring hospitalization with a total score ≤ 1, 55 (51%) were diagnosed with digestive disease, including 10 with gastrointestinal bleeding, 5 with cholangitis, 6 with pancreatitis and 7 with appendicitis. This contrasts with the overall proportion of digestive disease of 19%. In addition, 16 patients (15%) were diagnosed with psychiatric disease and 12 (7%) with cerebral disease. This compares with overall proportions of 7% for psychiatric disease and 19% for cerebral disease. Only 14 patients were not hospitalized despite a high total score (≥ 11). These included 4 patients suffering from a hypoglycemic attack, 7 with loss of consciousness and suspected vasovagal syncope, 2 with atrial arrhythmia and 1 with malignancy. Among the 165 patients who died in the emergency room, one had a total score of 4, three scored 7, one scored 8, seven scored 9 and the remainder scored ≥ 10. Those patients scoring 4–9 rapidly underwent cardiopulmonary arrest following headache, chest pain, dyspnea, fatigue and loss of consciousness.

**Figure 3:**
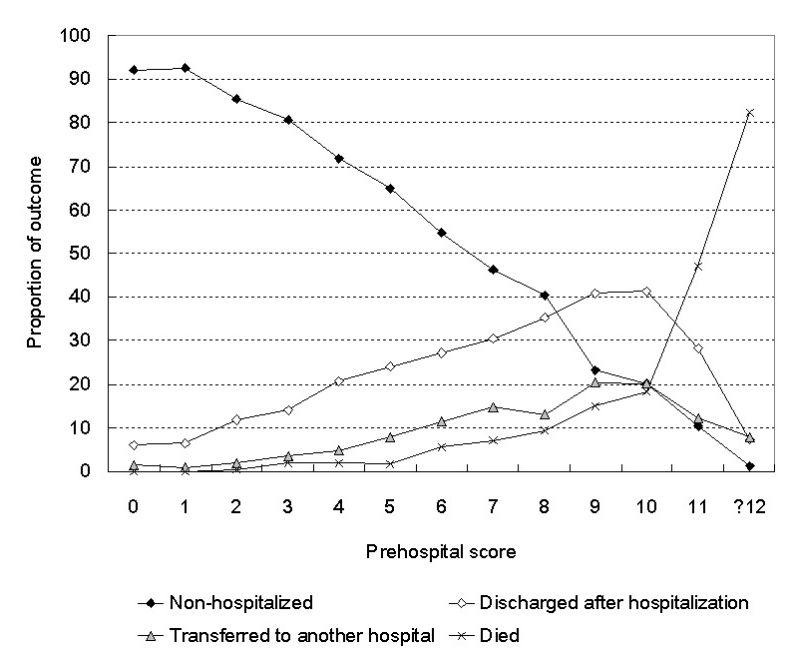
**Outcome proportions in relation to prehospital score**. The outcome proportions including after hospitalization were calculated for patients transported to the two main hospitals. The proportion discharged after hospitalization was higher than the proportion who died at scores ≤ 10, and *vice versa *at scores ≥ 11 (n = 6,498).

Figure 3 shows the proportions of patients transported to the two main hospitals who were non-hospitalized, discharged after hospitalization, transferred to another hospital or died (n = 6,498, 71% of the subjects). The proportion non-hospitalized declined, and the proportions discharged after hospitalization, transferred to another hospital or died all increased linearly, between scores 2 and 10. At score 0 or 1, there were no deaths and the proportion non-hospitalized was over 90%. The proportion discharged after hospitalization was higher than the proportion who died at scores ≤ 10. The proportion who died increased rapidly at scores ≥ 11 and exceeded 80% at scores ≥ 12. Among these 6,498 patients, 14 with total scores ≤ 3 died after hospitalization, including 7 with malignancy, 3 with liver cirrhosis and 2 with cardiovascular disease.

### Sensitivity and specificity of prehospital score

**Figure 4:**
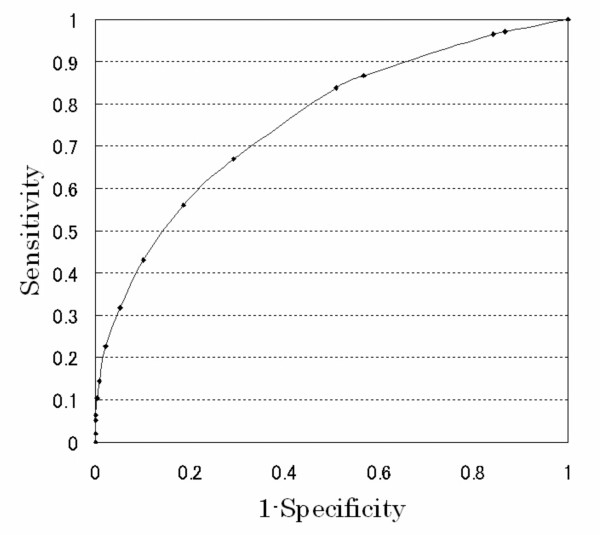
**ROC curve for prehospital score to predict hospitalization**. The area under the curve was 0.75 (95% confidence intervals 0.74–0.76, n = 8,330).

Figure 4 shows the ROC curve; the area under the curve is 0.75 (95% confidence interval 0.74–0.76, n = 8,330). When that was set at ≥ 2, the sensitivity, specificity, PPV and NPV were 97%, 16%, 39% and 89%, respectively (Table 2).

**Table 2:** Screening parameters according to each cut-off point of prehospital score

Cut off point	%Sensitivity	%Specificity	%PPV	%NPV
>=1	97.1	13.4	38.7	89.1
>=2	96.5	15.9	39.3	89.0
>=3	86.6	43.2	46.2	85.1
>=4	83.4	50.0	48.3	84.1
>=5	67.0	70.8	56.4	79.2
>=6	55.5	81.8	63.2	76.5
>=7	42.8	89.9	70.6	73.6
>=8	31.3	95.1	78.2	71.1
>=9	22.2	97.9	85.8	69.1
>=10	14.1	99.3	91.8	67.2
>=11	10.1	99.9	95.6	66.3
>=12	6.0	100	98.9	65.4
>=13	5.0	100	100	65.1
>=14	2.0	100	100	64.4

PPV: Positive predictive value

NPV: Negative predictive value

CI: Confidence interval

## Discussion

This study is an investigation of the relationship between prehospital data and requirement for hospitalization in acute disease patients aged ≥ 15 years; it also details the development of a simple triage assessment tool. Age, systolic blood pressure, pulse rate, level of consciousness, SpO_2_level and ability to walk were found to be statistically significant predictors for hospitalization requirement. A prehospital score ranging from 0 to 14 was developed on the basis of the multivariable analysis results.

In western countries, prehospital triage guidelines are based on patient symptoms for acute disease [10-16], but these are inappropriate for use in Japan as emergency life-saving technicians can only perform limited medical treatments.

There has been only one prehospital triage tool for acute disease and trauma in Japan: the prehospital severity and urgency criterion, produced by the Japan Foundation for Ambulance Service Development in 2004 [17]. However, this criterion is complex to use because it is based on 10 categories: trauma, burn, intoxication, consciousness disorder, chest pain, dyspnea, gastrointestinal bleeding, abdominal pain, pregnancy, and infancy; it requires approximately 20 items to be checked. In practice, this criterion is not used because of its complexity. Moreover, criteria based on chief complaints are of little use for cases with complaints outside those 10 categories, such as paralysis or headache [11].

In contrast, the prehospital score of the present study is a simple to use, comprehensive triage tool for acute disease. Furthermore, emergency personnel without emergency life-saving technician licences could use this scoring system because of its simplicity.

Sensitivity, specificity, PPV and NPV are barometers for the utility of a triage tool. As under-triage is less desirable than over-triage, sensitivity is more important than specificity. When we set the cut-off point at total score ≥ 2, sensitivity and specificity were respectively 97% (2,900/3,002) and 16% (841/5,328). Furthermore, we can also predict the likelihood of death as 0% and 100% when the score is low (≤ 1) or high (≥ 12), respectively.

If ambulance transfer is refused for patients with score ≤ 1, 16% (841/5,328) of cases of inappropriate ambulance usage would be avoided. On the other hand, this decision made 3.5% (102/3,002) into under-triage cases.

Previous studies in western countries have reported prehospital guidelines for predicting the requirement for admission to the emergency department (sensitivity = 90% and specificity = 37%) [11] and a prehospital protocol for predicting the critical event during transport (sensitivity = 95% and specificity = 33%) [12]. These studies relied on detailed protocols according to symptoms. Although simple comparison with our study is difficult because of differences in the medical systems, our scoring system showed higher sensitivity and lower specificity. To avoid under-triage, lower specificity might be inevitable.

However, this study is not without its limitations. First, the scoring system did not include information other than the six fundamental elements. For example, the chief complaint is often strongly associated with outcome: chest pain, paralysis, hematemesis or melena. Patients with gastrointestinal bleeding usually require hospitalization, yet 10 patients in this study with gastrointestinal bleeding scored ≤ 1. Moreover, 4 cerebral infarction cases, 1 myocardial infarction case, 1 pneumothorax case and 1 tuberculosis case scored ≤ 1. These cases needed rapid transfer to an emergency hospital. If low-scoring (<=1) patients with chest pain, paralysis, hematemesis or melena were considered as indicating ambulance usage, 17% (17/102) of under-triage cases could be avoided. Since our purpose is to develop a simple triage tool that would be useful for ambulance refusal for Japan emergency personnel regardless of emergency life-saving technician licence, an additional assessment for chief complaints would be necessary for practical use. However, sensitivity and specificity did not change substantially when these chief complaints were taken into account (97% and 15%, respectively).

Second, the scoring system was mainly based on patient vital signs, but did not include body temperature or respiration rate. The method for measuring body temperature in the prehospital setting is not standardized; some are measured by tympanic temperature, others by axillary temperature. Therefore, we did not use body temperature. The inclusion of respiration rate in the regression model excluded 15% of the subject data and did not improve the predictive value for hospitalization (sensitivity = 97% and specificity = 15%).

Third, the proportion of emergency hospitalization in Kishiwada City is substantially lower than the national average: 36% compared with 49%. Criteria for hospitalization vary according to areas and hospitals. Furthermore, we could not obtain complete data for 9% of patients in Kishiwada City. Therefore, the external validity of the prehospital score is uncertain.

Fourth, we had no follow-up data on patients who were not hospitalized. Some might be misdiagnosed in the emergency room and not hospitalized at that time, but would then be hospitalized after a few days.

Despite these limitations, the present study suggests that a simple score based on 6 fundamental elements enables us to decide whether ambulance transfer is indicated with 97% sensitivity and 16% specificity. In the near future, refusal of ambulance transfer for mild patients may be allowed in Japan. Our prehospital score could be a tool for deciding transfer refusal by emergency personnel.

## Conclusion

We have developed a prehospital score, a simple acute disease triage tool for Japan's emergency medical service system. Further research on validity would be necessary.

## Competing interests

The author(s) declare that they have no competing interests.

## Authors' contributions

YM, HT and HF were involved in the acquisition of data. YT was involved in data analysis and drafting the manuscript. TT and HI provided statistical advice on study design and critical revision of the manuscript. All authors read and approved the final manuscript

## Pre-publication history

The pre-publication history for this paper can be accessed here:



## References

[B1] Circumstances of Emergency and Rescue. Agency ofFire and Disaster Management. http://www.fdma.go.jp/neuter/topics/statistcs/pdf/h17_kyukyu_kyujo.pdf.

[B2] Lewin MR, Hori S, Aikawa N (2005). Emergency medical services in Japan: an opportunity for the rational development of pre-hospital care and research. J Emerg Med.

[B3] Pozner CN, Zane R, Nelson SJ, Levine M (2004). International EMS Systems: The United States: past, present, and future. Resuscitation.

[B4] Adnet F, Lapostolle (2004). International EMS Systems: France. Resuscitation.

[B5] Black JJM, Davies GD (2005). International EMS Systems: United Kingdom. Resuscitation.

[B6] Health and Welfare Statistics Association (2005). Population Statistics. Kosei No Shihyo.

[B7] Ohshige K, Mizushima S, Watanabe J, Mukasa M, Kawano T, Sekiguchi T, Awashima T, Tochikubo O (2001). Utilization of emergency ambulances in Yokohama city, Japan. Nippon Koshu Eisei Zasshi.

[B8] Ohsgige K, Tochikubo O (2003). A descriptive study on the trend of ambulance utilization in an aging society, Yokohama, Japan. Yokohama Med Bull.

[B9] Satou O, Yata K (1996). Consciousness disorder. Hyojun-Noshinkei-Gekagaku.

[B10] Schmidt T, Atcheson R, Federiuk C, Mann NC, Pinney T, Fullaer D, Colbry K (2000). Evaluation of protocols allowing emergency medical technicians to determine need for treatment and transport. Acad Emerg Med.

[B11] Pointer JE, Levitt MA, Young JC, Promes SB, Messana BJ, Ader MEJ (2001). Can paramedics using guidelines accurately triage patients?. Ann Emerg Med.

[B12] Holstein A, Plaschke A, Vogel MY, Egberts EH (2003). Prehospital management of diabetic emergencies-a population-based intervention study. Acta Anaesthesiol Scand.

[B13] Tirschwell DL, Longstreth WT, Becker KJ, Gammans RE, Sabounjian LA, Hamilton S, Morgenstern LB (2002). Shortening the NIH stroke scale for use in the prehospital setting. Stroke.

[B14] Llanes JN, Kidwell CS, Starkman S, Leary MC, Eckstein M, Saver JL (2004). The Los Angeles Motor Scale (LAMS): a new measure to characterize stroke severity in the field. Prehosp Emerg Care.

[B15] Welsh RC, Ornato J, Armstrong PW (2003). Prehospital management of acute ST-elevation myocardial infarction: A time for reappraisal in North America. American Heart J.

[B16] McVaney KE, Macht M, Clowell CB, Pons PT (2005). Treatment of suspected cardiac ischemia with aspirin by paramedics in an urban emergency medical services system. Prehosp Emerg Care.

[B17] The prehospital severity and urgency criteria. Foundation of Ambulance Service Development. http://www.fasd.or.jp/tyousa/hanso01.pdf.

